# Unravelling the Genetic Structure of Local and Mainstream Red‐Pied Cattle Breeds Using Genomics and Extended Pedigree Analysis

**DOI:** 10.1111/jbg.70054

**Published:** 2026-05-27

**Authors:** Margaux Vandewalle, Renzo Bonifazi, Jérémie Vandenplas, Sipke‐Joost Hiemstra, Gerben de Jong, Dirk Hinrichs, Nicolas Gengler, Hélène Wilmot

**Affiliations:** ^1^ TERRA Teaching and Research Centre, Gembloux Agro‐Bio Tech University of Liège Gembloux Belgium; ^2^ Animal Breeding and Genomics Wageningen University & Research Wageningen the Netherlands; ^3^ Centre for Genetic Resources, the Netherlands (CGN) Wageningen University & Research Wageningen the Netherlands; ^4^ Coöperatie Koninklijke CRV u.a Arnhem the Netherlands; ^5^ Animal Breeding Section, Faculty of Organic Agricultural Sciences University of Kassel Witzenhausen Germany; ^6^ Department of Animal and Dairy Science University of Georgia Athens USA

**Keywords:** gene flow, genetic diversity, genomic analysis, inbreeding, local endangered breeds

## Abstract

During the last decade, there has been a growing interest for local and endangered breeds as they are often seen as more resilient and healthier than mainstream breeds. They can also provide high added‐value products like meat or dairy products. To better preserve these breeds, it is of main importance to characterize their genetic diversity and have an insight of historical gene flow with more mainstream breeds. In this study, we focused on the genetic relationships of five red‐pied cattle breeds: the east Belgian red and white (EBRW); the red‐pied of the Ösling (RPO), from Luxembourg; the deep red (DR) and the Meuse‐Rhine‐Yssel (MRY), both from the Netherlands; and the German red and white dual purpose (RDN). The EBRW, RPO and DR breeds have an official European endangered status. We first investigated the pedigree completeness of available genotyped animals as well as their complex historical relationships through the analysis of common ancestors. We also compared pedigree and SNP‐based inbreeding coefficients, defined as the sum of homozygosity‐by‐descent segments (HBD). We then dived into genomic relationships through a classical multi‐dimensional scaling (MDS) of genotyped animals and their admixture. The pedigree analyses showed the complex gene flow between all breeds and that the RPO breed was the most connected to other breeds. Results also showed that the level of inbreeding was so far not an issue in all five breeds even if some animals, for example, in EBRW and MRY breeds, showed higher inbreeding levels than the average of their breeds. Finally, the MDS and admixture analysis also highlighted complex gene flow between the studied breeds and that they may be considered as a genetic continuum. This genomic proximity has the potential to improve genetic evaluations of local breeds by the inclusion of information from more mainstream breeds like MRY and RDN.

**Abbreviations**
DR: deep red, locally known as Brandrood
EBRW: east Belgian red and white, locally known as Rouge‐Pie de l'Est/Ostbelgische Rotbunte
ecg: equivalent complete generation
fullGen: full number of generations traced back
HBD: homozygous‐by‐descent
MDS: multidimensional scaling
MRY: Meuse‐Rhine‐Yssel
pc_g_
: pedigree completeness of generation g
PCI: pedigree completeness index
RDN: German red and White dual‐purpose, locally known as Rotbunte DN
RPO: red‐pied of the Ösling, locally known as Pie‐Rouge de l'Ösling/Öslinger Rotbunte
SNP: single nucleotide polymorphism

## 1 Introduction

The industrialization of agriculture and the increasing demand for productive livestock, especially after the Second World War, led to the marginalization or crossbreeding of many local dual‐purpose cattle breeds in Europe with specialized breeds to enhance milk or meat production (Hiemstra et al. 2010). This has led to reduced genetic diversity up to the extinction of entire breeds in the most severe cases (Hiemstra et al. 2010). However, preserving local dual‐purpose cattle breeds remains essential for several reasons. In addition to contributing to cultural heritage, these breeds sometimes generate high‐quality, local products with a strong territorial anchoring. These products often have a higher added value, appeal to consumers and help to compensate for the generally lower productivity of the breeds (Marsoner et al. 2018). Additionally, local breeds are often well adapted to their environments and to available resources, making them more efficient in those conditions, especially in extensive or low‐input farming systems (Hoffmann 2010; Liu et al. 2025). Because they have not been intensively selected for production traits, local breeds tend to be more robust, with better disease resistance and physical resilience. This can lead to reduced veterinary costs, although outcomes also depend on farm management practices, such as organic farming (Curone et al. 2018; Bieber et al. 2019). Importantly, local breeds represent a valuable reservoir of genetic material, acting as a safeguard for current and future challenges in animal breeding (Hoffmann 2010; Sponenberg et al. 2019; Jacques et al. 2023). Dual‐purpose breeds therefore remain relevant today for their flexibility, economic stability and sustainability, as they can supply both dairy and beef markets and contribute to lower greenhouse gas emissions per unit of output (Bay et al. 2010a; Bieber et al. 2019; Zanon et al. 2023).

In North‐Western Europe, a continuum of lowland, red‐pied dual‐purpose breeds stretches across France, Belgium, Luxembourg, the Netherlands and Germany, with several of them being currently endangered (Gengler and Wilmot 2022). Past studies suggest that, despite some gene flow from specialised breeds, many of these dual‐purpose breeds have retained their unique characteristics (François et al. 2017; Schmidtmann et al. 2021; Wilmot et al. 2023). To help their conservation, it is essential to assess both within‐ and between‐breed genetic diversity, which allows for strategies to monitor inbreeding, maintain breed differentiation and evaluate the feasibility of genetic exchanges—such as through semen or importation of animals—to support endangered populations (Bennewitz et al. 2008; Sponenberg et al. 2019).

In this study, we focused on five lowland red‐pied dual‐purpose breeds: the east Belgian red and white (EBRW) from Belgium, the deep red (DR) from the Netherlands, the red‐pied of the Ösling (RPO) from Luxembourg (all three endangered) and two more mainstream breeds, the Meuse‐Rhine‐Yssel (MRY) from the Netherlands and the German Red and White dual‐purpose breed (RDN) from Germany. The objective of this study is to characterise the between‐ and within‐breed diversity of these five breeds by the analysis of pedigree, inbreeding, classical multi‐dimensional scaling and admixture. To our knowledge, this is the first study to jointly analyse these five breeds, despite historical and ongoing exchanges between them. Moreover, while previous studies focused on genomic data (Marjanovic et al. 2021; Schmidtmann et al. 2021; Wilmot et al. 2023), we also performed an extended study of pedigree overlaps among these breeds, to highlight their historical connections. The hypothesis is that these breeds are historically connected and that smaller, endangered breeds may benefit from future collaborations with other related dual‐purpose red‐pied breeds.

## 2 Materials and Methods

### 2.1 Data

Genotypes from five red‐pied cattle breeds were included in this study: EBRW (*n* = 349), MRY (*n* = 279), DR (*n* = 49), RDN (*n* = 65) and RPO (*n* = 175). The genotypes from EBRW were provided by the Walloon Breeders' association (Elevéo; Belgium), those from MRY and DR by the Centre for Genetic Resources, the Netherlands (CGN), those from RPO by Convis (Luxemburg) and those from RDN by the Rinderzucht Schleswig‐Holstein (RSH) breeding company (Germany). Countries of origin from each breed are detailed in Table 1. The different chips used for genotyping were the Bovine SNP50 BeadChip v2 and v3, the EuroG MD v2, v3, v4, v4‐1 and v9‐SI. Distribution of genotypes for each chip type, breed and sex can be found in Table S1. Genotypes from MRY and RDN were considered representative samples of bulls used for artificial insemination (AI). For MRY, representative samples of semen from AI bulls have been collected since the 1960's and semen from all AI bulls have been collected since the 1990's. For EBRW, there were more genotyped males, while there were more genotyped females in RPO, reflecting the specific registration practices of their respective herdbook. Genotypes from RPO came from the same herd, which is the only one with officially recognized RPO animals. More details about the herdbook legislation of EBRW and RPO can be accessed in Wilmot et al. (2022). To ensure that the same SNPs across different genotype chips point to the same physical positions on the genome, all the SNPs from all available chips were mapped to the ARS‐UCD1.2 assembly (Rosen et al. 2020). Then, SNPs with a call rate below 0.9 within each breed were excluded. The remaining missing SNPs were imputed separately within each breed using Beagle 5.3 (Browning et al. 2018). The genotypes were imputed only within‐breed to avoid the introduction of bias from across‐breed imputation that would make local breeds more similar to mainstream ones and potentially hinder the interpretation of the results. Moreover, there is no crossbred available to do the across‐breed imputation. We kept SNPs at the overlap of the different SNP chips after imputation, leading to a final dataset of 27,807 SNPs. This relatively low number of SNPs is related to the fact that imputation was done within‐breed and that different SNP chips were used across breeds. Finally, to prevent the loss of SNPs that are (nearly) fixed in local breeds, we did not apply a minor allele frequency filter, because our objective was to investigate the differentiation among local and mainstream breeds.

**TABLE 1:** Number of animals in the pedigree of genotyped animals; Mean number of generations traced in the pedigree for genotyped animals (fullGen; rounded); Mean equivalent complete generations (ecg); Proportion of animals in the pedigree with a pedigree completeness index (PCI) of at least 0.6; Average and maximum pedigree‐based inbreeding coefficient for genotyped animals with a PCI of at least 0.6; Threshold *T* for rates *R*_k_ obtained as the smallest value greater than or equal to twice the mean number of generations in the pedigree; Average and maximum SNP‐based inbreeding coefficient computed as cumulated homozygosity over HBD classes with a rate *R*_k_ ≤ *T*; Pearson correlation between pedigree‐based and SNP‐based inbreeding coefficient for genotyped animals with PCI ≥ 0.6.

Breed	Country	Number of animals in the pedigree	Mean fullGen (rounded)	Mean ecg	% of animals with PCI ≥ 0.6^a^	Average pedigree‐based inbreeding coefficient (PCI ≥ 0.6)	Maximum pedigree‐based inbreeding coefficient (PCI ≥ 0.6)	Threshold T	Average SNP‐based inbreeding coefficient	Maximum SNP‐based inbreeding coefficient	Pearson correlation (PCI ≥ 0.6)
DR (*n* = 49)	The Netherlands	74	1	0.33	0.0	NA	NA	2	0.001	0.053	NA
EBRW (*n* = 349)	Belgium	5841	6	1.67	12.9	0.007	0.125	16	0.020	0.245	0.354
MRY (*n* = 279)	The Netherlands	3153	15	5.29	84.2	0.029	0.145	32	0.080	0.246	0.585
RDN (*n* = 65)	Germany	2645	14	4.57	63.1	0.026	0.082	32	0.058	0.140	0.645
RPO (*n* = 175)	Luxembourg	5179	26	7.92	90.3	0.027	0.176	64	0.049	0.142	0.395

Abbreviations: DR, deep red; EBRW, east Belgian red and white; MRY, Meuse‐Rhine‐Yssel; RDN, German red and white dual‐purpose; RPO, red‐pied of the Ösling.

^a^ PCI threshold defined as in Li et al. (2009).

Pedigree information was available for all breeds except RDN. Pedigree information was provided by Elevéo (Belgium) for EBRW and by Convis (Luxemburg) for RPO. Pedigree for MRY and DR were obtained from CGN and corrected or completed with pedigree obtained from the CRV website (the Netherlands; https://cooperatie‐crv.nl/zoek‐stier/). As a result, pedigree information was only available for bulls with published breeding values. A common pedigree was built using the R package *pedigree* (version 1.4.2) by merging all available pedigree and pruning branches of the final pedigree that did not contain genotyped animals, that is, only pedigree of genotyped animals was kept. Although no dedicated RDN pedigree was available, some RDN bulls appeared in the Belgian, Dutch, or Luxembourgish pedigree, providing partial pedigree information for some RDN. The final common pedigree included 9720 individuals. As breed information was not provided in the pedigree data, only animals with genotypic data were assigned to a breed. We therefore focused on genotyped animals in this study, even for pedigree analyses.

### 2.2 Common Ancestors of Genotyped Animals

Common ancestors among genotyped animals were identified by first creating a breed‐specific pedigree. The breed‐specific pedigree was obtained by extracting all the ancestors of the genotyped animals of a specific breed from the full pedigree. To find common ancestors between two breeds, we looked at the overlap between their respective breed‐specific pedigrees. Finally, for each genotyped individual, the pedigree distance from these common ancestors was calculated by counting the smallest number of generations separating them. The goal was to make a pairwise comparison of the distribution of the number of generations separating genotyped animals from common ancestors. The same procedure was applied to the intersection of all breed‐specific pedigrees to identify common ancestors shared across all five breeds, rather than only pairwise.

### 2.3 Pedigree Completeness

Pedigree completeness was evaluated in four ways for genotyped animals within each breed. All approaches used functions from the R package *optiSel* v.2.0.9 (Wellmann 2019). The first method calculated for each breed the mean pedigree completeness for generation *g* (pc_g_):

$${\mathrm{pc}}_{g}=\frac{1}{n}\sum_{i=1}^{n}a_{g_{i}}$$

where n is the number of genotyped animals in the breed, and $a_{g_{i}}$ is the proportion of known ancestors in generation g for genotyped animal i.

The second method estimated the number of equivalent complete generations (ecg) for each animal *i*, defined as the cumulative proportion of known ancestors across all generations:

$${\mathrm{ecg}}_{i}=\sum_{g=0}^{d}a_{g}$$

where d is the pedigree depth of that animal *i*, and $a_{g}$ is its proportion of known ancestors in generation g. The resulting values were then averaged across all genotyped individuals within each breed.

The third method was the full number of generations (fullGen) traced for each individual, also averaged within each breed. This method gives information about the pedigree depth of each breed and should be related to its completeness.

The fourth approach involved the pedigree completeness index (PCI), recommended by Wellmann (2019) to identify animals with insufficient pedigree information for inbreeding estimation. PCI is defined as the harmonic mean of the parents' pedigree completeness and was originally proposed by MacCluer et al. (1983). In *optiSel*, PCI is implemented according to Wellmann (2019) by first computing the parental index as follows:

$$I_{\mathrm{par}}=\frac{1}{d}\sum_{i=1}^{d}a_{i}$$

where $I_{\mathrm{par}}$ is the parental index of pedigree completeness, defined as a function of d, the considered pedigree depth (fixed to four generations by Wellmann 2019) and $a_{i}$, the proportion of known ancestors in generation i. Then, once the maternal and paternal index, $I_{p}$ and $I_{p}$ respectively, are defined, PCI can be calculated as follows:

$$\mathrm{PCI}=\frac{2\times I_{m}\times I_{p}}{I_{m}+I_{p}}$$

These four methods were chosen to provide a comprehensive assessment of pedigree completeness for each breed: pc_g_ captures generation‐specific information, ecg reflects overall ancestral knowledge, fullGen gives a direct measure of pedigree depth and PCI was used to filter individuals with insufficient pedigree data for inbreeding estimation.

### 2.4 Inbreeding

Inbreeding coefficients were calculated using the R package *pedigree* v.1.4.2 (Coster 2022) using all available generations of the pedigree of genotyped animals from all breeds. Given that the pedigree for the five breeds was incomplete for many animals, pedigree‐based inbreeding coefficients were only computed for animals with a PCI greater than or equal to 0.6, as defined by Li et al. (2009), as animals with low pedigree completeness may lead to an underestimation of pedigree‐based inbreeding.

In addition to pedigree‐based estimates, inbreeding coefficients can also be derived from genotypic data. SNP‐based inbreeding coefficients were estimated using homozygous‐by‐descent (HBD) segments, which are stretches of homozygosity (i.e., runs of homozygosity) inherited from a common ancestor shared by both parents. By analysing the length and the rate of these segments, it is possible to assess individual inbreeding level and to determine how recent the inbreeding event is (Bertrand et al. 2019). The R‐package *RZooRoH* (version 0.3.2.1), adapted for low‐coverage datasets, was used to identify HBD segments and to partition them into HBD classes that are related to the age of the common ancestor contributing to the individual autozygosity (Bertrand et al. 2019). The model was built including one non‐HBD class and nine HBD classes, each defined by a base rate of 2. This means that for each HBD class k from 1 to 9, the rate R_k_ was calculated as 2^k^, resulting in the values: 2, 4, 8, 16, 32, 64, 128, 256 and 512. Each rate R_k_ approximately corresponds to twice the number of generations separating the individual from the common ancestor contributing to autozygosity, and the physical length is 1/R_k_ Morgans (Bertrand et al. 2019). By using a base rate of 2 we created more HBD classes for recent ancestors. SNP‐based inbreeding coefficients may then be obtained by cumulating homozygosity over HBD classes with a rate R_k_ lower or equal to a defined threshold *T*. The threshold *T* for *R*_k_ was set separately for each breed, as the smallest value greater than or equal to twice the average number of generations in the corresponding breed‐specific pedigree (Table 1). The number of cumulated HBD classes can be interpreted as different base populations extending up to 0.5*T generations ago (Solé et al. 2017). The probability of a heterozygous pair in the HBD segment was set to the default value of 0.001.

Pearson correlations between pedigree‐based and SNP‐based inbreeding coefficients were calculated for genotyped animals. Again, to ensure a reliable comparison with pedigree‐based estimates, the computation of Pearson correlations was restricted to genotyped individuals with a PCI of at least 0.6.

### 2.5 Classical Multidimensional Scaling

A classical multidimensional scaling (MDS) was performed to assess genomic relatedness between the different breeds. Prior to the MDS, linkage disequilibrium pruning was performed using PLINK v.1.9 (Chang et al. 2015) with the option ‘‐‐indep‐pairwise 50 5 0.8’ (Wilmot et al. 2023), resulting in a final dataset of 26,501 SNPs. A distance matrix was computed with PLINK v.1.9 (option ‘‐‐distance‐matrix’), and classical MDS was applied in R with the *cmdscale()* function. The first three dimensions were visualized using R v.4.5.0 (R Core Team 2022). Additional dimensions were not included, as they did not improve breed separation and the proportion of explained variance reached a plateau (Figure S1).

### 2.6 Admixture

To complete the analysis of genomic relatedness, an admixture analysis was conducted with ADMIXTURE v.1.3 (Alexander et al. 2009). To avoid bias due to relationships between individuals, we performed a two‐step selection of genotyped animals. The first step allows the selection of the lowest related animals within each breed while the second step pooled all breeds together to select the lowest related set of animals across breeds. This two‐step procedure was applied as some animals are used across breeds, but most animals are only related to animals from their own breed. We used the R package SNPRelate v.1.44.0 (Zheng et al. 2012) to compute a genomic relationship matrix (GRM), excluding SNPs with minor allele frequency lower than 0.05. We then computed the average genomic relationship of each animal with animals from their own breed and excluding self‐relationships. In the first step, we created a breed‐specific pool. For each breed, we started a pool including the least related animals by selecting the animal with the lowest average relationship with all other animals of the same breed. We then iteratively added animals of the same breed that had the lowest average relationship with this pool. Animals were added to the pool only if their average genomic relationship with the pool was lower than 0.05. Each pool included at least 25 animals from the same breed and at most 120 animals. These minimum and maximum values of 25 and 120 animals were used to ensure having balanced samples across breeds for ADMIXTURE. We relaxed the threshold of average genomic relationship of 0.05 for the DR breed to obtain the minimum number of 25 animals for the ADMIXTURE analysis.

In the second step, we created a global pool by merging all the breed's pools selected in the first step. We first selected the animal with the lowest global average relationship (i.e., with all animals from all breeds), and then iteratively added animals with the lowest average relationship with the global selected pool. During this process, we ensured that each breed was still represented by a minimum of 25 animals. We arbitrary stopped the global pool when 400 animals were reached. Using this set of 400 animals, we pruned the genotypes as described in the previous section (option ‘‐‐indep‐pairwise 50 5 0.8’, leading to 26,501 SNPs) and finally ran the ADMIXTURE analysis. The number of clusters analysed ranged from one to five, corresponding to the number of breeds included in the study. To evaluate the optimized number of clusters, we looked at the error of a 10‐fold cross‐validation. The belonging of individuals to each of the clusters was visualized using R v.4.5.0 (R Core Team 2022).

## 3 Results

### 3.1 Pedigree Completeness

**FIGURE 1:**
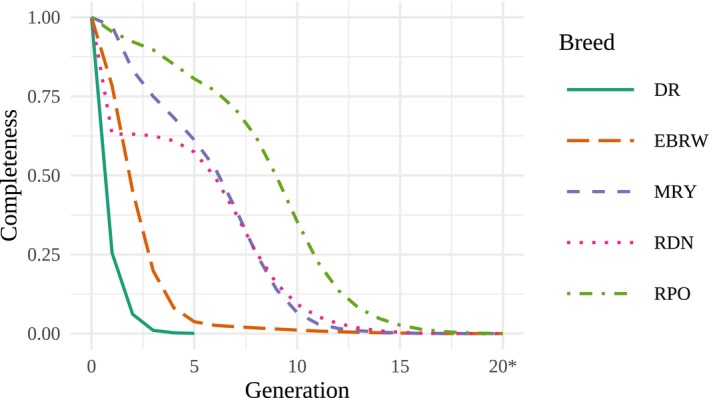
Pedigree completeness as the average proportion of known ancestors per generation (pc_g_). *The maximum number of generations was 30, but the *x*‐axis is limited to 20 for improved readability. DR, deep red; EBRW, east Belgian red and white; MRY, Meuse‐Rhine‐Yssel; RDN, German Red and White dual‐purpose; RPO, red‐pied of the Ösling. [Colour figure can be viewed at wileyonlinelibrary.com]

Pedigree completeness for genotyped animals was evaluated using four complementary methods, which yielded consistent results across approaches. Figure 1 illustrates the first measure of pedigree completeness (pc_g_), defined as the proportion of known ancestors across generations while Table 1 details results obtained on average for fullGen and ecg as well as the percentage of animals with PCI ≥ 0.6. Table S2 shows, for each breed, the average, minimum and maximum number of fullGen traced back as well as the proportion of animals with 0, 1 or 2 known parents. RPO animals had the highest pedigree completeness, as also shown by the greatest proportion of individuals achieving a PCI above 0.6 and the highest ecg (Table 1). In contrast, DR animals had the least complete pedigree. Of the 49 DR individuals, 34 completely lacked pedigree information (i.e., both parents were unknown). While the maximum pedigree depth observed in this group was five generations, the corresponding PCI did not exceed 0.6, indicating that only few ancestors were known across generations. Consequently, no DR animals met the PCI threshold of 0.6 required for inclusion in pedigree‐based inbreeding coefficient calculations. Despite genotyped EBRW animals having the largest overall pedigree size (Table 1), a substantial proportion of them had incomplete pedigree. Only 13% of the 349 genotyped EBRW animals had a PCI above 0.6. Notably, 140 individuals lacked information for at least one parent, contributing to a low PCI. Some EBRW animals displayed extensive pedigree depth (up to 28 generations) yet retained low completeness, suggesting the presence of one or a few long but sparsely documented ancestral lines. For example, one individual had a pedigree depth of 28 generations but a PCI of 0 as the paternal line was completely unknown. Among RDN animals, 24 out of 65 had no pedigree information, whereas the remaining 41 individuals had more complete pedigree and therefore PCI above the 0.6 threshold. This pattern is reflected in Figure 1, where a marked drop in mean pedigree completeness is observed in the first generation due to the presence of animals with unknown ancestry. MRY animals also demonstrated relatively good pedigree completeness: only 12 of the 279 MRY individuals lacked information for one or both parents.

### 3.2 Common Ancestors of Genotyped Animals

**FIGURE 2:**
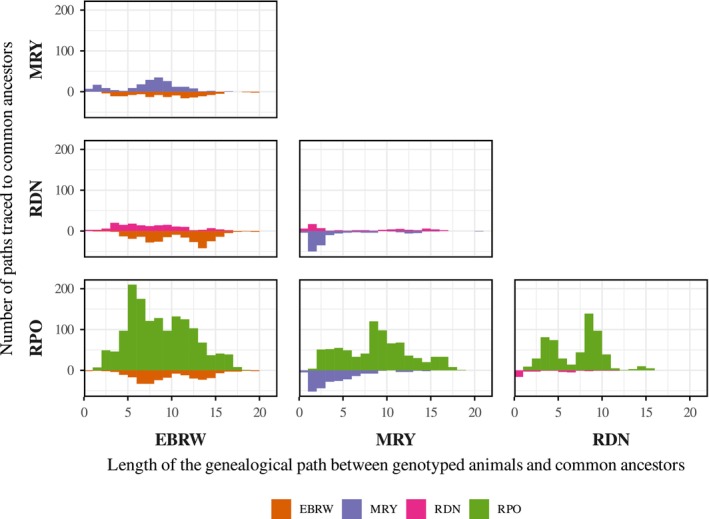
Pairwise distributions of the number of generations separating genotyped animals from common ancestors between two breeds. Genealogical path = upward sequence of offspring‐to‐parent links connecting a genotyped animal to its common ancestor shared with another breed. EBRW, east Belgian red and white; MRY, Meuse‐Rhine‐Yssel; RDN, German Red and White dual‐purpose; RPO, red‐pied of the Ösling. [Colour figure can be viewed at wileyonlinelibrary.com]

The number of pairwise common ancestors identified among each pair of breeds is presented in Table 2. Due to incomplete pedigree data for DR animals, no common ancestors could be identified between this breed and the four others. After these common ancestors were identified, genealogical paths linking genotyped animals to their common ancestors with other breeds in the pedigree were traced back. As a reminder, a genealogical path was defined as the upward sequence of offspring‐to‐parent links connecting a genotyped animal to its common ancestor shared with another breed. The distribution of these paths by their length (i.e., the number of generations separating a genotyped individual from its common ancestor) is shown in Figure 2. A path length of zero indicates that a genotyped animal is itself the common ancestor shared with another breed. As one animal can be linked to multiple common ancestors and a common ancestor can be linked to multiple genotyped animals, the total number of paths traced between breed pairs may vary. RPO animals exhibited the highest number of genealogical connections to common ancestors with other breeds (Figure S2). As shown in Figure 2, most of these links were ancient, with only a few cases involving common ancestors, one generation away (i.e., a parent). MRY animals appeared to have more recent common ancestors with RPO and RDN animals, with many common ancestors being the parents or grandparents of genotyped MRY individuals. In some cases, genotyped MRY animals were themselves common ancestors of other breeds as EBRW and RDN. In contrast, the links between EBRW and the other breeds were generally more ancient. It is important to interpret these results in the light of Figure S3, which shows that genotyped MRY and RDN animals were generally older than EBRW and RPO animals. This difference in birth years affects the comparison of genealogical path lengths across breeds. Notably, RDN genotyped animals spanned the same birth years as many MRY animals, although the MRY population also included older genotyped individuals going back up to the 1960s. This suggests that the shared ancestors of MRY and RDN genotyped animals were occurring in recent generations (Figure 2). However, the corresponding generations were born earlier than those of EBRW and RPO genotyped animals, which were generally younger (Figure S3).

**TABLE 2:** Number of common ancestors between each pair of breeds.

	EBRW	MRY	RDN	RPO
MRY	39			
RDN	51	35		
RPO	74	39	33	
DR	0	0	0	0

Abbreviations: DR, deep red; EBRW, east Belgian red and white; MRY, Meuse‐Rhine‐Yssel; RDN, German Red and White dual‐purpose; RPO, red‐pied of the Ösling.

**FIGURE 3:**
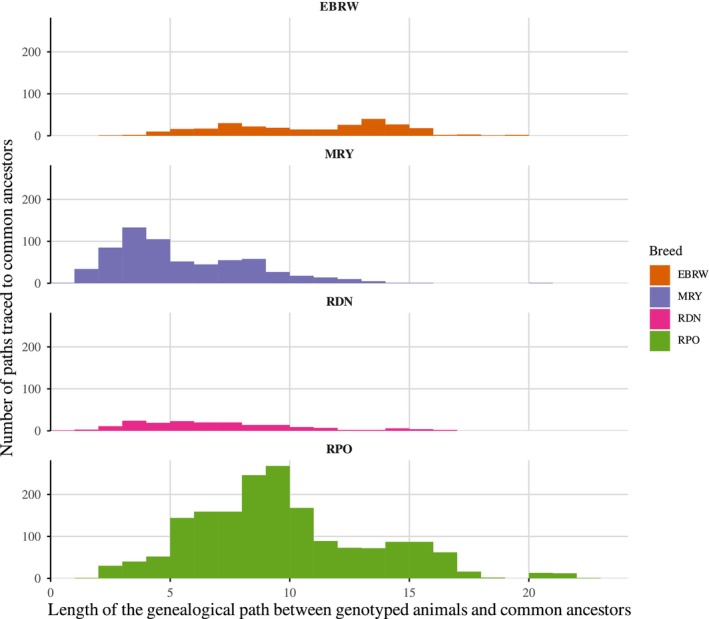
Distribution of the number of generations separating genotyped animals from the common ancestors between EBRW, MRY, RDN and RPO. Genealogical path = upward sequence of offspring‐to‐parent links connecting a genotyped animal to its common ancestor shared across all breeds. Total number of paths: EBRW = 266, MRY = 645, RDN = 181, RPO = 1781. EBRW, east Belgian red and white; MRY, Meuse‐Rhine‐Yssel; RDN, German red and white dual‐purpose; RPO, red‐pied of the Ösling. [Colour figure can be viewed at wileyonlinelibrary.com]

When comparing the separate pedigree of EBRW, MRY, RPO and RDN, excluding DR, 44 common ancestors were identified across all four breeds. These common ancestors were generally present in more recent generations for RDN and MRY genotyped animals than in RPO and EBRW animals (Figure 3), which is also due to the difference in birth years of genotyped animals. Notably, one genotyped MRY bull and one genotyped RDN bull were identified as common ancestors of all four breeds.

### 3.3 Pedigree‐Based Inbreeding

On average, pedigree‐based inbreeding coefficients were low across all breeds (Table 1), although not all animals could be included in the analysis due to a PCI below 0.6. EBRW exhibited the lowest average inbreeding coefficient. The highest individual inbreeding levels observed for EBRW, MRY and RPO animals were primarily the result of half‐sib matings, often involving parents that themselves were already inbred, leading to inbreeding coefficients exceeding 0.125. A noteworthy case involved one DR animal with an inbreeding coefficient of 0.25, resulting from a sire‐daughter mating. However, due to an incomplete maternal lineage, this animal's PCI was too low for the inbreeding coefficient to be included in the analysis.

### 3.4 SNP‐Based Inbreeding

**FIGURE 4:**
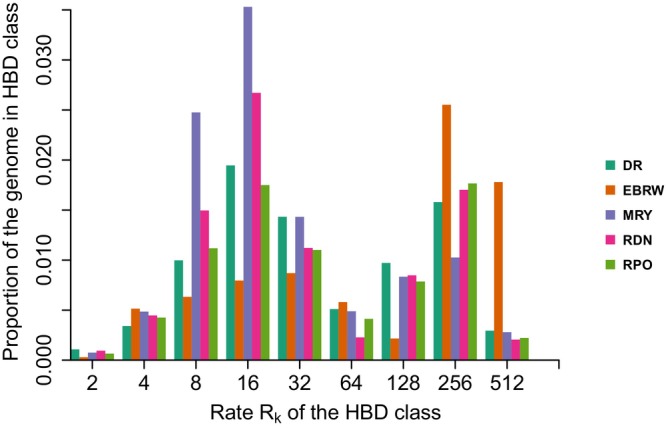
Proportion of the genome in different homozygous‐by‐descent (HBD) classes defined by rates R_k_. DR, deep red; EBRW, east Belgian red and white; MRY, Meuse‐Rhine‐Yssel; RDN, German red and white dual‐purpose; RPO, red‐pied of the Ösling. [Colour figure can be viewed at wileyonlinelibrary.com]

Figure 4 shows the proportion of the genome assigned to each HBD class defined by rates *R*_k_. Overall, these proportions were relatively low across all breeds and rates. MRY animals exhibited higher proportions at rates *R*_k_ = 8 and *R*_k_ = 16 compared to the other breeds, indicating common ancestors approximately 4 and 8 generations ago between these animals. RDN animals also showed a higher proportion at *R*_k_ = 16 compared to other rates. At *R*_k_ = 256, proportions increased across all breeds and at *R*_k_ = 512, EBRW animals displayed higher values than other breeds. However, inbreeding events at these higher rates reflect very ancient common ancestry, even before breeds were created.

**FIGURE 5:**
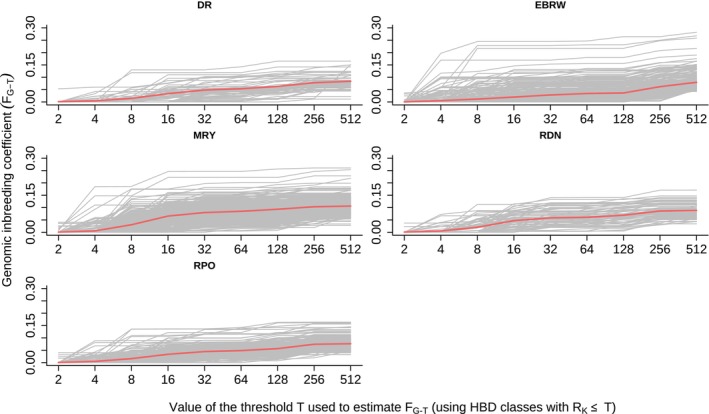
SNP‐based inbreeding coefficients computed as cumulated homozygosity over homozygous‐by‐descent (HBD) classes with a rate *R*_k_ ≤ *T*. Grey = individual SNP‐based inbreeding coefficient; red = average SNP‐based inbreeding coefficient. DR, deep red; EBRW, east Belgian red and white; MRY, Meuse‐Rhine‐Yssel; RDN, German red and white dual‐purpose; RPO, red‐pied of the Ösling. [Colour figure can be viewed at wileyonlinelibrary.com]

Figure 5 presents the average SNP‐based inbreeding accumulated at each rate R_k_. At *R*_k_ = 512, average inbreeding coefficients remained below 0.12 for all breeds. Individual SNP‐based inbreeding coefficients revealed limited variability among DR, RDN and RPO animals. In contrast, some MRY and especially EBRW individuals displayed higher levels of inbreeding: three EBRW animals had SNP‐based inbreeding coefficients exceeding 0.2 at *R*_k_ = 16, and two MRY animals exceeded 0.2 at *R*_k_ = 32. The pedigree of the three EBRW animals was too incomplete to detect any inbreeding event that could explain the high SNP‐based inbreeding coefficient. For MRY, only one of the two animals had a sufficiently complete pedigree (PCI = 0.97), with a pedigree‐based inbreeding coefficient of 0.14 resulting from a half‐sib mating, compared to a SNP‐based inbreeding coefficient of 0.25, suggesting undetected inbreeding events in the recorded pedigree.

Table 1 reports SNP‐based inbreeding coefficient at a specific threshold T for each breed. Although the coefficients for DR animals may appear low, the chosen threshold T was also low due to the very incomplete pedigree (Table 1). Nonetheless, even at a threshold T = 64, which is the maximum threshold used across all breeds, the average inbreeding coefficient for DR animals was 0.084, showing a SNP‐based inbreeding comparable to the levels observed in the other breeds.

The Pearson correlation between pedigree‐based and SNP‐based inbreeding coefficients was moderate for MRY and RDN, yet higher than for the other breeds (Table 1). RPO showed a lower correlation compared to other breeds despite having the most complete pedigree. In general, SNP‐based inbreeding coefficients were higher than pedigree‐based ones for all breeds. An exception was observed in eight RPO animals and one EBRW animal, for which the pedigree‐based inbreeding coefficient was more than twice as high as the genomic‐based inbreeding coefficient, highlighting incorrect pedigree recording for these animals.

### 3.5 Classical Multidimensional Scaling

**FIGURE 6:**
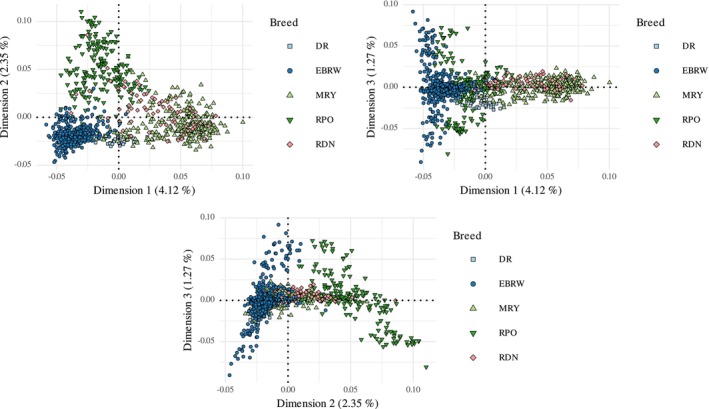
First three dimensions of the classical multidimensional scaling analysis. DR, deep red; EBRW, east Belgian red and white; MRY, Meuse‐Rhine‐Yssel; RDN, German Red and White dual‐purpose; RPO, red‐pied of the Ösling. [Colour figure can be viewed at wileyonlinelibrary.com]

The first three dimensions from the classical multidimensional scaling analysis explained 4.12%, 2.35% and 1.27% of the total variation, respectively (Figure 6). The breeds did not form clearly distinct clusters along any of these axes, but rather showed a continuum, reflecting close genetic relationships. Nonetheless, some patterns of differentiation were observed. Along the first dimension, a gradient was visible from EBRW to DR and then to MRY. The second dimension highlighted a separation of RPO from the other breeds, although some individuals overlapped with EBRW, MRY and to a greater extent, RDN. RDN and MRY appeared genetically similar, showing substantial overlap and little separation across all three dimensions.

### 3.6 Admixture

**FIGURE 7:**
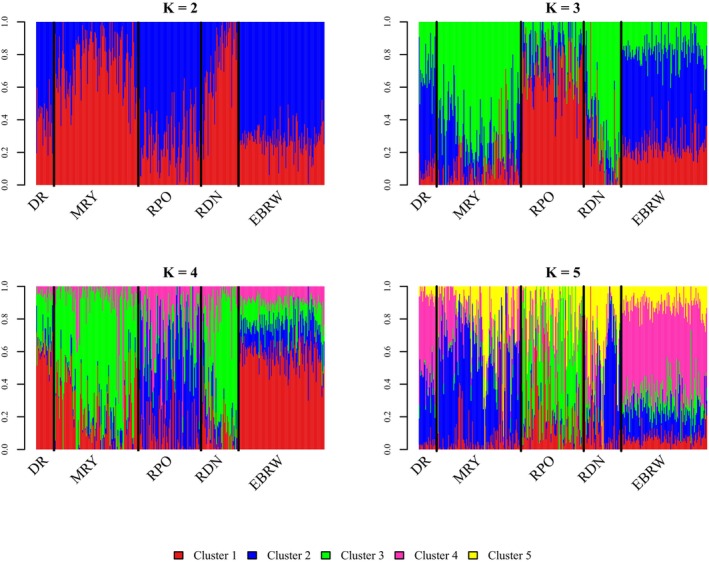
Clusters *K* = 2–5 obtained with the ADMIXTURE software. DR, deep red; EBRW, east Belgian red and white; MRY, Meuse‐Rhine‐Yssel; RDN, German red and white dual‐purpose; RPO, red‐pied of the Ösling. [Colour figure can be viewed at wileyonlinelibrary.com]

Following the two‐step procedure described above to select the less related animals, a total of 25 DR, 119 EBRW, 117 MRY, 52 RDN and 87 RPO were available for the ADMIXTURE analysis. Figure 7 shows the results of the admixture analysis for cluster numbers *K* = 2 to *K* = 5. As the cross‐validation error did not stabilize at any value of *K* with a 10‐fold cross‐validation, the optimized number of clusters was selected based on the number of breeds. At *K* = 2, a separation was already visible, with MRY and RDN forming one group and EBRW, DR and RPO forming another. At *K* = 3, RPO and EBRW began to differentiate from each other and from the other breeds. Across all tested values of K, MRY consistently appeared distinct from EBRW, while MRY and RDN remained more or less clustered. This agrees with the first dimension of the MDS (Figure 6). DR seems to share ancestry fractions with both MRY and EBRW, regardless of *K*.

## 4 Discussion

This study aimed to evaluate the genetic diversity and relatedness of five red‐pied lowland dual‐purpose cattle breeds (DR, EBRW, MRY, RDN and RPO) using both SNP data and available pedigree information. To our knowledge, this is the first study to jointly analyse these specific breeds with both genomic and pedigree data. While these breeds are genetically related, their differences were also reflected in the results. The MDS and the admixture analyses showed that these breeds formed a genetic continuum even if the EBRW still clusters apart. Nonetheless, previous studies already showed that correct assignment is possible with a high accuracy for animals from these five breeds (Wilmot et al. 2022). The study of the pedigree showed that the RPO breed seemed more connected to other breeds, at different generations and that MRY has more complete and reliable pedigree than local breeds. This is in line with the fact that MRY herdbook keeping has been continuous and self‐contained for decades while exporting many animals. The pedigree of RPO while deep seemed to be more prone to errors than MRY. RPO breed has been incorporating many imported animals on a large scale into a less maintained population. This could be a reason that pedigree is less precise for RPO. For all breeds, inbreeding does not seem to be a current issue, but it should still be monitored to prevent any increase. Despite we only had genotypes from AI bulls for MRY and RDN breeds, our results agree with previous studies that used larger datasets for MRY or RDN (Marjanovic et al. 2021; Schmidtmann et al. 2021). Moreover, as it is usually bulls from mainstream breeds (here MRY and RPO) that are (historically) imported in local herds (DR, EBRW and RPO), we expect that the data available is a representative sample to detect genetic relationships between those breeds.

### 4.1 Pedigree Completeness and Implications

A major limitation in the pedigree analysis was its incompleteness, particularly for DR and EBRW, or even its unavailability for RDN. Indeed, pedigree information for RDN was available only through the pedigree of the other four breeds. As such, 24 genotyped RDN bulls lacked known ancestry, suggesting no common ancestry with the other breeds. However, this is unlikely given that these 24 bulls clustered similarly to the other RDN bulls in the classical MDS and ADMIXTURE analyses. Therefore, their lack of pedigree information is most likely due to incomplete records in the available pedigree. Historically, when AI bulls were used across countries, their pedigree was typically transferred only on paper for two generations. Electronic exchanges of pedigree between national databases were later established but mostly in Holstein or other major breeds. Therefore, the incomplete pedigree of these RDN bulls may indicate that their pedigree information was not properly transferred to countries of exportation. In contrast, pedigree completeness for MRY was high, explained by the fact that nearly all genotyped MRY bulls (96%) had published breeding values, which requires a sufficiently known pedigree. The absence of published breeding values for most DR animals aligns with their incomplete pedigree and reflects the broader reality of poor pedigree recording in this breed (CGN 2009).

The RPO population, sampled from the only farm registered in the herdbook, showed many individuals tracing back to the same ancestors. This pattern is more likely a result of a more complete pedigree compared to other breeds, rather than evidence of a closed population that may lead to inbreeding. As shown by our results, inbreeding did not seem to be a problem so far in the RPO population. Differences in pedigree depth and completeness across breeds can bias the assessment of common ancestry, so caution is needed when interpreting these results.

### 4.2 Inbreeding: Pedigree and Genomic Estimates

Pedigree‐based inbreeding coefficients were generally low. However, the vast majority of EBRW animals and all DR animals could not be evaluated because of incomplete pedigree. Although a PCI threshold of 0.6 was applied, pedigree‐based inbreeding was likely underestimated because of a remaining degree of incompleteness (Lutaaya et al. 1999). Consequently, animals showing already high inbreeding values may, in fact, be even more inbred. Therefore, SNP‐based inbreeding estimates were also calculated. SNP‐based inbreeding coefficients were also low and consistent with estimates for these breeds in previous studies (Eynard et al. 2018; Schmidtmann et al. 2021; Wilmot et al. 2023). The few EBRW and MRY animals showing SNP‐based inbreeding levels exceeding 0.2 underscored how incomplete pedigree can mask substantial inbreeding. Similar to the results observed in this study, Schmidtmann et al. (2021) also found that MRY animals had a higher proportion of the genome associated with smaller HBD classes compared to RDN, though both breeds stay well under the levels observed in other North European red‐pied breeds not included in this study (Schmidtmann et al. 2021). The higher homozygosity observed in MRY and RDN may reflect more intensive selection compared to EBRW, DR and RPO breeds, which lack well‐established breeding programs or are less strongly selected. Additionally, due to their smaller population sizes, these latter three breeds are more frequently crossed with other red‐pied breeds to maintain genetic diversity.

The higher Pearson correlation between pedigree‐based and SNP‐based inbreeding coefficients for MRY and RDN compared to RPO, despite its high PCI values, is likely due to the official pedigree recording systems in place for the former two breeds, which are instead lacking in RPO, making MRY and RDN less prone to pedigree errors. Once again, the importance of genomic information for a better estimation of inbreeding levels is highlighted in small local breeds.

### 4.3 Genetic Relatedness and Historical Exchanges

The percentages of explained variation for the MDS were similar to those obtained by a previous study, based on some of the same breeds (4.29%, 3.45% and 1.60% for the first three principal components in Wilmot et al. 2023). These low percentages can be explained by low between‐breed variation. MDS and admixture analyses (Figure 6, Figure 7) confirmed a certain degree of clustering between breeds while also revealing genetic exchanges. As expected, MRY and RDN showed close genetic proximity, consistent with known historical connections despite their distinct geographical origins (Schmidtmann et al. 2021). It is also known that MRY and RDN can be distinguished based on a genomic breed assignment tool (Wilmot et al. 2022). Our analyses also suggested shared ancestry between DR and the other breeds, although this cannot be confirmed by pedigree due to missing information. The DR breed originated from MRY after the Second World War, when breeders aimed to preserve the deep red coat and muscularity that were neglected in favour of higher milk production in MRY (CGN 2009). Decades later, in the late 2000s, EBRW animals were introduced into the DR population to increase genetic diversity during a period of near extinction (Vereniging Het Brandrode Rund 2025). These exchanges likely explain the strong genetic proximity between DR and EBRW, which have a stronger selection for beef traits compared to the more dairy‐oriented MRY (CGN 2009; Bay et al. 2010b). Nonetheless, genetic links between MRY and DR remain visible in both genomic analyses, as also previously shown in Wilmot et al. (2023).

A previous study reported genetic contributions from MRY in RPO (Wilmot et al. 2023). However, in our analysis, RPO is already clearly differentiated from MRY at *K* = 2 in the admixture analysis (Figure 7). Pedigree analysis revealed that nearly all the most recent common ancestors shared between RPO and MRY were of German origin. In the absence of breed labels in the pedigree, these ancestors were likely RDN animals. Wilmot et al. (2025) also reported that RPO animals shared genomic ancestry with MRY animals born within the past decade, a period coinciding with increased exchanges between the MRY and RDN populations. The possible genetic relationship between RPO and MRY observed in earlier studies is therefore more likely the result of indirect introgression of MRY genetics through RDN bulls used in the RPO herd (Wilmot et al. 2025). Further evidence of a close relationship between RPO and RDN is visible in Figure 6, where some RDN bulls cluster closely with RPO animals. To date, only one other study has compared RPO and RDN (Wilmot et al. 2022), mainly with the objective to assign properly animals to their respective breeds with a genomic assignment tool. Despite the small sample size (17 RDN animals), RPO individuals also appeared genetically close to RDN in Wilmot et al. (2022). This pattern is consistent with breeding practices in the RPO herd, where RDN bulls are regularly introduced to maintain genetic diversity and EBRW animals are occasionally used (Wilmot 2021; Wilmot et al. 2023).

### 4.4 Conservation and Breeding Perspectives

Despite reduced population sizes, endangered breeds such as EBRW, DR and RPO maintain considerable within‐breed diversity. This is likely due to gene flow from other red‐pied breeds like MRY and RDN at various paces, following legislation and breeding objectives during the last decades as detailed in Wilmot et al. (2025). These exchanges also managed to maintain between‐breed diversity as observed in figures 6 and 7 and shown in Wilmot et al. (2022), illustrating that gene flow between related breeds can successfully preserve both within‐ and between‐breed diversity (Bennewitz et al. 2008). However, as already explained in previous studies (e.g., Wilmot et al. 2025), the exchange of animals and/or semen between dual‐purpose red‐pied breeds is sub‐optimal so far, as breeders usually rely on the phenotype of the foreign animal (i.e., their coat colour or how much they look like the ideal animal from the targeted breed) to make a choice of importation. The development of genomic breed assignment tools as in Wilmot et al. (2022) allows to determine if the animal to be imported is close enough to the targeted breed. These tools also have an important role in the recent implementation of official pedigree monitoring programmes in endangered breeds. The pedigree of local breeds such as DR, EBRW and RPO may still contain a higher rate of incomplete or inaccurate records compared to those of MRY and RDN. Regarding the derogation of article 19 of the European regulation 2016/1012 that allows the inclusion of animals without pedigree in the main section of the herdbook of an endangered breed, genomic breed assignment tools are therefore very helpful, allowing breeders to obtain related subsidies.

Another problem related to missing and incomplete pedigree is the accuracy of selection for poorly recorded breeds. Although genomic evaluations are becoming increasingly common, national genetic evaluations may still rely on pedigree information for predicting breeding values. The development of multibreed genomic evaluation systems has therefore been proposed to enable more reliable selection (Schmidtmann et al. 2021; Wilmot et al. 2023). Such genomic evaluation systems do not require pedigree information, but a reference multibreed population of sufficient size. However, genomic predictions depend on linkage disequilibrium (LD) between SNPs and QTLs, which varies across breeds (Schmidtmann et al. 2021). Consequently, adding animals from other breeds may not provide the same accuracy as increasing the reference population within the breed of interest (Marjanovic et al. 2021). In addition, limited data hinder the estimation of QTL effects which can vary across breeds (Hayes et al. 2009; Schmidtmann et al. 2021). Another strategy to improve genomic predictions is the integration of foreign breeding values from related red‐pied breeds, such as MRY and RDN, into the national genetic evaluation of endangered breeds, for example, by using a Bayesian integration model (i.e., foreign breeding values and reliabilities are used as priors) (Vandenplas et al. 2014). These breeds benefit from reliable breeding values and structured breeding programs, making them suitable sources for such prior information. Sparse pedigree data could be combined with genomic information to create a combined genomic‐ and pedigree‐based relationship matrix (Aguilar et al. 2010). The integration of reliable foreign breeding values would make use of the genetic links established via the combined relationship matrix. This would also improve the decisions of breeders to import an animal or semen by providing EBVs from foreign animals that are integrated into the local system.

To optimise further exchange of animals/semen between dual‐purpose red‐pied breeds, limit inbreeding and preserve the intrinsic genetic diversity of breeds, breeding circles could be used (Windig and Kaal 2008; Windig et al. 2019). Each year several sires would migrate from a donor to a recipient population in a circular way. We could also imagine going one step further and pooling local and mainstream dual‐purpose red‐pied breeds to implement a two‐step breeding circle. In the first step, animals would be exchanged between the local red‐pied breeds (DR, EBRW and RPO). In a second step, this pool of local breeds would be used to import animals from MRY and RDN breeds, at a lower rate than migration occurring in the first step and therefore preserving the intrinsic specificities of local breeds.

## 5 Conclusions

The present study showed the complex genetic and historical relationships between five local red‐pied cattle breeds: the DR, EBRW, MRY, RDN and RPO, from four different countries (Belgium, Germany, Luxembourg and the Netherlands). The study of the pedigree showed many common ancestors at different generations in the breeds involved. As expected, pedigree recording was more complete and reliable in mainstream breeds like MRY. Results of MDS and admixture showed that the five breeds formed a genetic continuum, reflecting past and ongoing gene exchanges. Even though inbreeding is not a current issue in these breeds, this parameter should still be monitored. Genetic exchanges can be a danger to breed distinctiveness. However, in the here described continuum of related breeds, optimized use of close animals can also be useful to preserve the distinctiveness of each breed and of the whole group. Local breeds might benefit from their genetic connections to MRY and RDN through the development of genetic evaluations including foreign information from MRY and RDN, which should be explored in further studies.

## Author Contributions

**Margaux Vandewalle:** conceptualization, methodology, software, formal analysis, investigation, writing – original draft, visualization; **Renzo Bonifazi:** conceptualization, methodology, investigation, writing – review and editing; **Jérémie Vandenplas:** conceptualization, methodology, software, investigation, writing – review and editing; **Sipke‐Joost Hiemstra:** investigation, resources, writing – review and editing; **Gerben de Jong:** investigation, resources, writing – review and editing; **Dirk Hinrichs:** investigation, resources, writing – review and editing; **Nicolas Gengler:** conceptualization, methodology, investigation, resources, writing – review and editing; **Hélène Wilmot:** conceptualization, methodology, software, investigation, writing – review and editing, supervision.

## Funding

This work was supported by Erasmus+. Direction Générale Opérationnelle Agriculture, Ressources Naturelles et Environnement du Service Public de Wallonie. Fonds De La Recherche Scientifique ‐ FNRS, T.0095.19, J.0174.18.

## Ethics Statement

The SNP data for the animals included in this study was previously obtained on samples collected by concerned breeder associations based on relevant authorisation by the different local authorities.

## Conflicts of Interest

The authors declare no conflicts of interest.

## Supporting information


**Table S1:** Total number of genotyped animals, number of genotyped males and females and distributions of bead chips used for genotyping each breed.
**Table S2:** Average, minimum and maximum number of generations (fullGen) traced per breed; Proportion of genotyped animals with 0, 1 or 2 known parents.
**Figure S1:** Proportion of the total variance explained by the first 10 dimensions of the muti‐dimensional scaling.
**Figure S2:** Total number of genealogical paths traced between genotyped animals and their common ancestor within each breed pair.
**Figure S3:** Distribution of birth years across breeds.

## Data Availability

The data supporting the findings of this study cannot be made available as a whole. The corresponding author, upon reasonable request, will forward the request to relevant data owners.
