# Supplementary material for: Unravelling the Genetic Structure of Local and Mainstream Red‐Pied Cattle Breeds Using Genomics and Extended Pedigree Analysis
Source: J Anim Breed Genet. 2026 May 27;143(5):705–18. doi: 10.1111/jbg.70054 (PMC13460504; doi:10.1111/jbg.70054)
Supplement: Supplementary file 1 [file JBG-143-705-s001.docx]

**SUPPLEMENTARY MATERIAL**

**Supplementary Table 1.** Total number of genotyped animals, number of genotyped males and females, and distributions of bead chips used for genotyping each breed.

| Breed | n animals | n males | n females | Bovine SNP50 v2 | Bovine SNP50 v3 | EuroG MD v2 | EuroG MD v3 | EuroG MD v4 | EuroG MD v4-1 | EuroG MD v9-SI |
| --- | --- | --- | --- | --- | --- | --- | --- | --- | --- | --- |
| DR | 49 | 24 | 25 | 7 | 42 | 0 | 0 | 0 | 0 | 0 |
| EBRW | 350 | 261 | 89 | 115 | 83 | 45 | 13 | 24 | 31 | 38 |
| MRY | 279 | 279 | 0 | 129 | 150 | 0 | 0 | 0 | 0 | 0 |
| RDN | 65 | 65 | 0 | 65 | 0 | 0 | 0 | 0 | 0 | 0 |
| RPO | 175 | 10 | 165 | 0 | 108 | 22 | 0 | 35 | 0 | 10 |

Abbreviations: DR = Deep Red, EBRW = East Belgian Red and White, MRY = Meuse-Rhine-Yssel, RDN = German Red and White dual-purpose, RPO = Red-Pied of the Ösling.

**Supplementary Table 2:** Average, minimum and maximum number of generations (fullGen) traced per breed; Proportion of genotyped animals with 0, 1 or 2 known parents

|  | Average of fullGen | Minimum  fullGen | Maximum fullGen | Proportion of animals with 0/1/2 known parents |
| --- | --- | --- | --- | --- |
| DR | 0.5 | 0 | 5 | 0.69 / 0.10 / 0.20 |
| EBRW | 6.0 | 0 | 28 | 0.03 / 0.37 / 0.60 |
| MRY | 14.6 | 0 | 28 | 0.01 / 0.03 / 0.96 |
| RDN | 13.3 | 0 | 24 | 0.37 / 0 / 0.63 |
| RPO | 26.1 | 0 | 30 | 0.01 / 0.08 / 0.91 |

Abbreviations: DR = Deep Red, EBRW = East Belgian Red and White, MRY = Meuse-Rhine-Yssel, RDN = German Red and White dual-purpose, RPO = Red-Pied of the Ösling.

**Supplementary Figure 1.** Proportion of the total variance explained by the first 10 dimensions of the muti-dimensional scaling.

**Supplementary Figure 2.** Total number of genealogical paths traced between genotyped animals and their common ancestor within each breed pair.

Abbreviations: DR = Deep Red, EBRW = East Belgian Red and White, MRY = Meuse-Rhine-Yssel, RDN = German Red and White dual-purpose, RPO = Red-Pied of the Ösling.

Note: Total number of paths across all combinations: EBRW = 645, MRY = 549, RDN = 266, RPO = 2861

**Supplementary Figure 3:** Distribution of birth years across breeds.

Abbreviations: DR = Deep Red, EBRW = East Belgian Red and White, MRY = Meuse-Rhine-Yssel, RDN = German Red and White dual-purpose, RPO = Red-Pied of the Ösling.

Note: Number of animals with unknown birth year: DR = 9, EBRW = 3, MRY = 0, RDN = 0, RPO = 1.
